# Interleukin 35-Producing Exosomes Suppress Neuroinflammation and Autoimmune Uveitis

**DOI:** 10.3389/fimmu.2020.01051

**Published:** 2020-05-29

**Authors:** Minkyung Kang, Jin Kyeong Choi, Yingyos Jittayasothorn, Charles E. Egwuagu

**Affiliations:** ^1^Molecular Immunology Section, Laboratory of Immunology, National Eye Institute (NEI), National Institute of Health, Bethesda, MD, United States; ^2^Department of Immunology, Jeonbuk National University Medical School, Jeonju, Jeonbuk, South Korea; ^3^Immunoregulation Section, Laboratory of Immunology, National Eye Institute (NEI), National Institute of Health, Bethesda, MD, United States

**Keywords:** exosome, regulatory B-cell, bregs, uveitis, experimental autoimmune uveitis (EAU)

## Abstract

Corticosteroids are effective therapy for autoimmune diseases but serious adverse effects preclude their prolonged use. However, immune-suppressive biologics that inhibit lymphoid proliferation are now in use as corticosteroid sparing-agents but with variable success; thus, the need to develop alternative immune-suppressive approaches including cell-based therapies. Efficacy of *ex-vivo*-generated IL-35-producing regulatory B-cells (i35-Bregs) in suppressing/ameliorating encephalomyelitis or uveitis in mouse models of multiple sclerosis or uveitis, respectively, is therefore a promising therapeutic approach for CNS autoimmune diseases. However, i35-Breg therapy in human uveitis would require producing autologous Bregs from each patient to avoid immune-rejection. Because exosomes exhibit minimal toxicity and immunogenicity, we investigated whether i35-Bregs release exosomes that can be exploited therapeutically. Here, we demonstrate that i35-Bregs release exosomes that contain IL-35 (i35-Exosomes). In this proof-of-concept study, we induced experimental autoimmune uveitis (EAU), monitored EAU progression by fundoscopy, histology, optical coherence tomography and electroretinography, and investigated whether i35-Exosomes treatment would suppress uveitis. Mice treated with i35-Exosomes developed mild EAU with low EAU scores and disease protection correlated with expansion of IL-10 and IL-35 secreting Treg cells with concomitant suppression of Th17 responses. In contrast, significant increase of Th17 cells in vitreous and retina of control mouse eyes was accompanied by severe choroiditis, massive retinal-folds, and photoreceptor cell damage. These hallmark features of severe uveitis were absent in exosome-treated mice and visual impairment detected by ERG was modest compared to control mice. Absence of toxicity or alloreactivity associated with exosomes thus makes i35-Exosomes attractive therapeutic option for delivering IL-35 into CNS tissues.

## Introduction

The CNS is an immune privileged site comprised of the brain, spinal cord and the ocular retina. Its intricate and highly vulnerable physiology is shielded from potentially pathogenic inflammatory cells by the blood-brain-barrier (BBB) or the blood-ocular-barrier (BOB) (1). Although resident ocular microglial cells or epithelial cells of the choroid plexus that constitutively secrete immunosuppressive cytokines contribute to the maintenance of immune privilege of the eye, brain or spinal cord, lymphocytes bearing antigen-receptors specific to oligodendrocytes or retinal proteins do breach the BBB or BOB during neuroinflammatory diseases, attack and destroy neurons, and photoreceptor cells (2). Thus, inflammation in the CNS presents unique challenges, as the need to avoid collateral damage that may compromise functional integrity of the retina or brain is as important as the need to eliminate the pathogen (2). It is now widely accepted that unrestrained neuroinflammation contributes to neuronal or photoreceptor cell deficit that precede neurodegenerative changes observed in chronic uveitis, multiple sclerosis, Alzheimer's disease, or age-related macular degeneration. Thus, inflammation in the CNS presents unique challenges, as the need to avoid collateral damage that may compromise neuro-retinal or brain functions has to be counterbalanced by the need to eliminate the pathogen (2). Although steroids are effective therapy for neuroinflammatory diseases, serious adverse effects preclude their prolonged use and effective long-term therapies remains an unmet medical need. Thus, there is considerable impetus to develop novel and effective alternative therapies such as biologics and cell-based therapies.

The IL-12 family cytokines have emerged as an important group of cytokines that regulate critical aspects of host immunity, including antigen presentation and T cell lineage commitment and they comprise of IL-12, IL-23, IL-27, and IL-35 and the newly described member, IL-39 (3, 4). IL-12, IL-23, and IL-39 promote development of chronic inflammatory diseases while IL-27 or IL-35 suppress inflammation and have been found to ameliorate uveitis or encephalitis in mice (5, 6). Although IL-35 or IL-27 show substantial promise as biologics for the treatment of autoimmune and neurodegenerative diseases, a major disadvantage of using these heterodimeric cytokines as biologics is their relatively short-half-life and unpredictable pharmacokinetic characteristics. However, recent reports have shown that IL-35-producing B cells (i35-Bregs) regulate immunity during CNS autoimmune diseases by inducing expansion of IL-10-producing Bregs (B10), IL-10-producing T cells (Tregs), and IL-35-producing T cells (iT_R_35) (7–9), suggesting that i35-Bregs can be exploited in treating autoimmune or infectious diseases. However, use of i35-Breg therapy in human uveitis is constrained by the need to produce autologous i35-Bregs for each uveitis patient in order to prevent immune-rejection of allogeneic i35-Bregs.

Exosomes are secreted by immune cells including lymphocytes and they contain proteins, lipids, nucleotides, miRNAs, and mRNAs (10–12). Their functions vary depending on the cell of origin and its physiological state. Exosomes modulate diverse cellular functions including proliferation, development, and metabolism by inducing differential mRNA expression in recipient cells, and in lymphocytes they mediate immune stimulation or immune suppression (12, 13). They are nanosized vesicles of 30–100 nm that can cross the BBB or BOB, deliver their cargo into the CNS, and can therefore be exploited in the treatment of neurogenerative and CNS autoimmune diseases (14, 15). In this study, we isolated exosomes from i35-Bregs and used them to treat uveitis.

## Materials and Methods

### Mice

Six- to eight-week-old C57BL/6J mice (were purchased from Jackson Laboratory (Jackson Laboratory, Bar Harbor, ME). Animal care and experimentation conformed to National Institutes of Health (NIH) guidelines and the experimental protocol was approved under NIH/NEI Animal Study Protocol (ASP) # NEI-597.

### Exosome Isolation and Characterization

Because i35-Bregs are mostly CD138^+^ plasma cells (16), splenic B cells isolated by use of MicroBeads from Miltenyi-Biotec (130-121-301) are used. For generation of i35-Breg exosomes (i35-Exosomes) the plasma cells were stimulation with anti-IgM/anti-CD40 Abs for 72 h at low density (<10^6^/ml). Analysis of aliquots for p35 and Ebi3 expression by flow cytometry, routinely showed i35-Breg enrichment (>35%) under this culture condition as previously described (7). Under this culture condition i35-Bregs or unstimulated CD19^+^ control B cells do not die as verified by Vi-Cell XR (Viability analyzer, Beckman Coulter, Indianapolis, IN). Complete media with exosome-depleted FBS was used for exosome isolation from culture supernatants of control and i35-Breg enriched cultures using Exoquick TC reagent (System Biosciences) following manufacturer's guidelines. Exosome size distribution was measured by Nanoparticle Tracking Analysis using the NanoSight system(NanoSight) and expression of exosome markers or IL-35 subunit proteins were characterized by Western blotting.

### Western Blotting Analysis

Exosomes were lysed in RIPA buffer [10 mM Tris-Cl (pH 8.0), 1 mM EDTA, 1% of Triton X-100, 0.1% sodium deoxycholate, 0.1% SDS, 140 mM NaCl, and 1 mM PMSF] and lysates were incubated for 30 min on 4°C. After incubation, lysates were centrifuged at 14,000 rpm for 30 min and supernatants were harvested. Lysates (7 μg/lane) were fractionated on 4–12% gradient SDS-PAGE, and antibodies used were: CD63, CD9, HSP70 (System Biosciences #EXOAB-KIT-1), p35 (Santa Cruz), and Ebi3 (Santa Cruz). After secondary antibodies reaction, signals were detected with LI-COR system (LI-COR Biosciences, Lincoln, NE). Image studio software (LI-COR Biosciences, Lincoln, NE) was used for data analysis.

### Immunoprecipitation

The i35-Exosome lysates were incubated with antibodies (4 μg of anti-Ebi3 or Normal IgG) overnight at 4°C. Next day, magnetic beads from Dynabeads Protein A Immunoprecipitation Kit (Thermo Fisher Scientific (Waltham, MA) were incubated with lysates for 1 h at 4°C and precipitated beads was washed and proteins were eluted and boiled for 10 min at 95°C. Samples were fractionated on 4–12% gradient SDS-PAGE and incubated with Ebi3 or p35 antibodies. After secondary antibodies reaction, signals were detected with LI-COR system. Image studio software was used for data analysis.

### CFSE (Carboxyfluorescein Succinimidyl) Dilution Assay

For CFSE dilution assay, cells were cultured for 72 h using a commercially available CFSE Cell Proliferation kit (Molecular Probes, Inc.). Graphical display showing information about cells undergoing various rounds of cell division was obtained from FlowJo software. The threshold of cellular proliferation was determined based on analysis of unstimulated cells.

### ELISA

CD4^+^ cells were isolated from spleen and lymph nodes by MACS cell separation system (Miltenyi, Cologne, Germany). For T cell activation, cells were seeded on the plates pre-coated with 3 μg/ml of anti-CD3 antibodies and incubated with 1 μg/ml soluble anti-CD28 antibodies and PBS or exosomes. After 24 h, we analyzed cytokines secreted in supernatant of the activated CD4^+^ T cells by Multiplex ELISA (R&D Systems, Minneapolis, MN).

### Experimental Autoimmune Uveitis (EAU)

EAU was induced by active immunization of C57BL/6J with IRBP_651−670_-peptide in a 0.2 ml emulsion (1:1 v/v with complete Freund's adjuvant (CFA) containing *Mycobacterium tuberculosis* strain H37RA (2.5 mg/ml). Mice also received *Bordetella pertussis* toxin (1 μg/mouse) concurrently with immunization (17). Mice were matched by age and sex and for most experiments 6–8 weeks mice were used (14 mice per group; *n* = *14*). Clinical disease was established and scored by fundoscopy and histology as described previously (7, 18, 19).

### Histology

Eyes for histology were enucleated, fixed in 10% buffered formalin, and serially sectioned in the vertical pupillary-optic nerve plane. Specimens are then dehydrated through graded alcohol series, embedded in methacrylate, serial transverse sections (4 μm) cut, and stained with hematoxylin and eosin (H&E). Photographs of representative sections are taken on a photomicroscope.

### Fundoscopy

Funduscopic examinations were performed at day 15 and 17 after EAU induction. Fundus image was captured using Micron III retinal imaging microscope (Phoenix Research Labs) for small rodent or a modified Karl Storz veterinary otoendoscope coupled with a Nikon D90 digital camera, as previously described (19, 20). To avoid a subjective bias was obviated by evaluating fundus photographs without knowledge of the mouse identity and by masked observers. At least six images (two posterior central retinal view, four peripheral retinal views) were taken from each eye by positioning the endoscope and viewing from superior, inferior, lateral, or medial fields, and each lesion was identified, mapped, and recorded. Clinical grading of retinal inflammation was as established (18, 21, 22).

### Optical Coherence Tomography (OCT)

Optical coherence tomography (OCT) is a non-invasive procedure that allows visualization of internal microstructure of various eye structures in living animals. Mice were then immobilized using adjustable holder that allow for horizontal or vertical scan scanning and each scan was performed at least twice, with realignment each time. The dimension of the scan (in depth and transverse extent) was adjusted until the optimal signal intensity and contrast was achieved. Retinal thickness was measured from the central retinal area of all images obtained from both horizontal and vertical scans from the same eye, using the system software, and averaged. The method used to determine the retinal thicknesses in the system software was as described (18, 23).

### Electroretinogram (ERG)

ERG measures changes in electrical potentials in response to light stimulation of the retina and is used to identify gross physiologic changes pathognomonic visual function defects. Before ERG recordings, mice are dark-adapted overnight, and experiments performed under dim red illumination. ERG is recorded on anesthetized mice using an electroretinography console that generates and controls the light stimulus. Dark-adapted ERG is recorded with single-flash delivered in a Ganzfeld dome and a reference electrode (gold wire) is placed in the mouth, and a ground electrode (subcutaneous stainless steel needle) is positioned at the base of the tail. Signals are differentially amplified and digitized. Amplitudes of the major ERG components (a- and b-wave) are measured by automated methods (18).

### Flow Cytometery

For intracellular cytokine detection, cells were restimulated for 4 h with PMA (20 ng/ml)/ionomycin (1 μM). GolgiStop was added in the last hour, and intracellular cytokine staining was performed using BD Biosciences Cytofix/Cytoperm kit as recommended (BD Pharmingen, San Diego, CA, USA). FACS analysis was performed on a MACSQuant analyzer (Miltenyi Biotec, San Diego, CA, USA) using protein-specific monoclonal antibodies and corresponding isotype control Abs (BD Pharmingen, San Diego, CA, USA) as described previously (9). FACS analysis was performed on samples stained with mAbs conjugated with fluorescent dyes, and each experiment was color-compensated. Dead cells were stained with dead cell exclusion dye (Fixable Viability Dye eFluor® 450; eBioscience), and live cells were subjected to side scatter and forward scatter analysis. Quadrant gates were set using isotype controls with <0.2% background.

### Statistical Analysis

Statistical analyses were performed by independent two-tailed Students's *t*-test. The data are presented as mean ± SEM.

## Results

### i35-Breg-Derived Exosomes Suppressed CD4^+^ T Cell Proliferation and INF-γ Secretion *in vitro*

**Figure 1:**
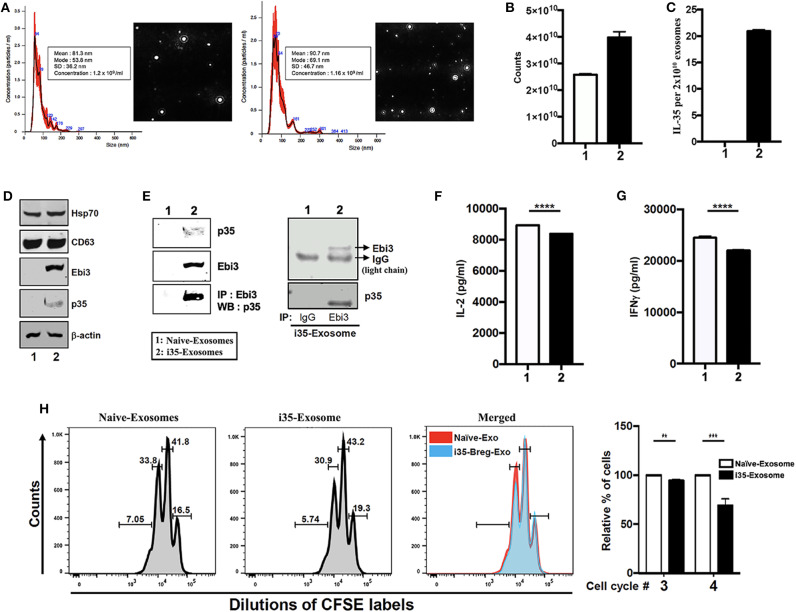
Breg cells release exosomes that contain the immune suppressive IL-35 cytokine. **(A)** Purification, characterization, and quantification of exosomes derived from activated B cells that do not produce IL-35 (Naïve-Exosomes) or IL-35-secreting regulatory B cells (i35-Exosomes). Size distribution analysis of exosome samples analyzed by Nanoparticle Tracking Analysis (NTA). Mean ± SEM of three independent experiments is shown. **(B,C)** Quantified of exosome released in unstimulated and stimulated B cell cultures by use of the Exosome Quantitation Assay **(B)** and determination of amounts of IL-35 contained in 2 × 10^10^ Naïve-Exosomes or i35-Exosomes (*n* = *6*) by ELISA **(C)**. **(D)** Western blot analysis of exosomal markers (HSP70 and CD63) expressed by exosomes derived from i35-Breg cells (right) or exosomes from B cells that do not produce IL-35 (Naive-Exosomes, left). **(E)** Lysates derived from Naïve-Exosomes or i35-Exosomes were subjected to immunoprecipitation/Western blot analysis using antibodies specific to Ebi3, p35 or mouse-IgG. **(F,G)** CD4^+^ T cells were stimulated *in vitro* for 3 days in culture medium containing anti-CD3/CD28 Abs and Naïve-Exosomes or i35-Exosomes. Secretion of IL-2 (F) or IFN-γ **(G)** in the supernatants was detected by ELISA (*n* = *6*). **(H)** CD4^+^ T cells were stimulated with anti-CD3/CD28 Abs for 4 days under non-polarizing condition in culture medium containing Naïve-Exosomes or i35-Exosomes (20 μg). Effect of Naïve-Exosomes or i35-Exosomes on lymphocyte proliferation was assessed by the CFSE dilution assay. Results represent three independent studies **p < 0.01, ***p < 0.001, ****p < 0.0001.

IL-35 produced by Breg cells plays critical roles in immunosuppression but instability of the IL-35 heterodimer (Ebi3/p35) relative to other IL-12 heterodimeric cytokines has impeded its clinical application (7). Reports of Treg-cell-mediated suppression by miRNA-containing exosomes and of exosomes derived from T cells that mediate Immune Responses (24, 25), suggested that i35-Bregs might secrete exosomes containing IL-35. To examine whether B cells can secrete IL-35 containing exosomes we seeded CD19^+^ B cells (1 × 10^6^ cells), stimulated the cells with anti-IgM/anti-CD40 for 72 h and pilot studies confirmed that IL-35-producing B cells (i35-Breg) were enriched in the culture (>35%) as previously described (7, 9). Control B cells were also cultured at 10^6^/ml and under this low density culture condition i35-Bregs or unstimulated CD19^+^ control B cells do not die. Exosome-enriched extracellular vesicles (EV) were isolated from the cell supernatant using ExoQuick exosome precipitation solution we used the Nanoparticle Tracking Analysis (NTA) method to determine particle size distribution of the exosomes which ranged from 50 to 150 nm for unstimulated CD19^+^ B cells (Naïve-Exosome) and Breg-derived exosomes (i35-Exosomes) (Figure 1A). Exosomes released in the culture were extracted from supernatants and exosome numbers was quantified using Exosome Quantitation Assay (System Biosciences). Average of 2.5 × 10^10^ and 4.0 × 10^10^ exosomes were secreted from unstimulated or stimulated CD19^+^ B cells, respectively (Figure 1B) and while exosomes from unstimulated B cells that did not produce IL-35 (Naïve-Exosomes), 2 × 10^10^ exosomes from i35-Exosomes were found to produce as much as 20 ng IL-35 (*n* = *6*) as determined by ELISA (Figure 1C). Western blot analysis of lysates prepared from the exosomes showed that both Naïve-Exosome and i35-Exosomes expressed the canonical exosome-markers CD63 and Hsp70 and confirmed that the Naïve-Exosomes did not express IL-35 while the i35-Exosomes secreted both p35 and Ebi3 subunits that associate to produce the heterodimeric IL-35 cytokine (Figure 1D). To provide direct evidence that the i35-Exosomes produce the heterodimeric IL-35, we performed reciprocal immunoprecipitation analysis using the antibodies specific to p35 and Ebi3. Precipitation of the extracts with the Ebi3 antibody and Western blot analysis using the p35 antibody confirmed the that the i35-Exosomes indeed produce the heterodimeric IL-35 (Figure 1E). Immunoprecipitation data showing equivalent IgG light-chain band intensities indicates that equal amount of total lysate was used for lanes 1 and 2 (Figure 1E, right panel). Because previous studies had shown that IL-35 suppresses effector functions of T cell (26, 27), we investigated whether the i35-Exosomes could inhibit capacity of T cells to produce effector cytokines in response to TCR activation. We isolated and purified naïve CD4^+^ cells from wild-type mice, stimulated the cells or 3 days in medium containing anti-CD3/anti-CD28 antibodies and Naïve-Exosome or i35-Exosome (1.27 × 10^10^ exosomes). Analysis of supernatant by ELISA assay showed that compared to cultures that received Naïve-Exosomes, i35-Exosome suppressed TCR-mediated secretion of IL-2 (Figure 1F) and IFN-γ (Figure 1G). We also examined the effects of i35-Exosomes on T cell proliferative response by the CFSE dilution assay. Significant inhibition of T cell proliferation by i35-Exosomes is consistent with the observed decrease of IL-2 and IFN-γ secretion (Figure 1H), underscoring efficacy of i35-Exosome in suppressing pro-inflammatory response of T cell *in vitro*.

### i35-Exosomes Suppressed Established Experimental Autoimmune Uveitis (EAU)

**Figure 2:**
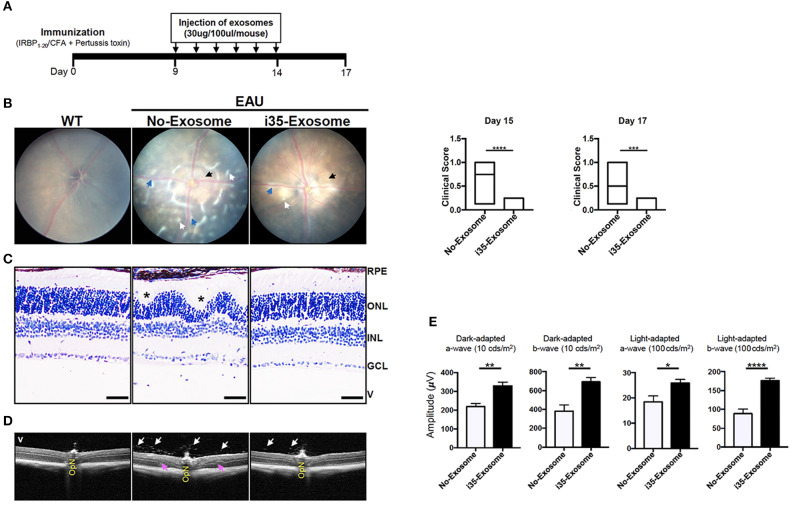
Mice treated with i35-Exosomes are protected from developing severe EAU. EAU was induced in C57BL/6J mice by immunization with the uveitogenic peptide, IRBP_651−670_ in CFA and effect of treatment with PBS or i35-Exosomes on EAU progression was assessed by fundoscopy, histology, optical coherence tomography (OCT), and electroretinography (ERG). **(A)** Scheme used for exosome treatment. **(B)** Fundus image of the retina was taken at day 15 or 17 after EAU induction using an otoendoscopic imaging system. Compared to mice treated with i35-Exosomes, fundus images of mice treated with PBS revealed more severe ocular inflammation characterized by significant blurring of the optic disc margins and enlarged juxtapupillary area (black arrow), retinal vasculitis (blue arrows), yellow-whitish retinal and choroidal infiltrates (white arrow). Clinical scores and assessment of disease severity were based on changes at the optic nerve disc or retinal vessels and retinal and choroidal infiltrates. Histogram to the right shows the clinical scores (*n* = *14*). **(C)** Histologic images. Eyes show very severe EAU in mice treated with PBS as characterized by the development massive retinal in-folding (*), a hallmark feature of severe uveitis. H&E histological sections: Scale bar, 100 μm. V, vitreous; GCL, ganglion cell layer; INL, inner nuclear layer; ONL, outer nuclear layer; RPE/CH retinal pigmented epithelial and choroid. Blue arrows, lymphocytes; Asterisks, retinal-folds. **(D)** Representative OCT images show marked decrease of inflammatory cells (white arrows) in the vitreous and optic nerve head of mice treated with i35-Exosomes (white arrows) **(E)** ERG analysis of the retina on day-17 after EAU induction. The averages of light- or dark-adapted ERG a-wave or b-wave amplitudes are plotted as a function of flash luminance and values are means ± SEM. Data are presented as the mean ± SEM of at least three determinations. Results represent three independent studies. *p < 0.05, **p < 0.01, ***p < 0.001, ****p < 0.0001.

In view of the immune-suppressive effect of i35-Exosomes *in vitro*, we investigated whether i35-Exosomes can be used to treat mice with experimental autoimmune uveitis (EAU), a model of human uveitis (28, 29). We induced EAU in C57BL/6J mice by active immunization with an autoantigenic peptide derived from interphotoreceptor retinoid-binding protein (IRBP_651−670_) in CFA emulsion (17). Mice were treated with ~2 × 10^10^ exosomes (30 μg/mouse) on day 9 post-immunization and every day until day 14 post-immunization by retro-orbital injection and disease severity was assessed on day-17 post-immunization. The immunization and exosome treatment strategy are shown (Figure 2A). Disease progression was monitored by fundoscopy, histology, optical coherence tomography, and electroretinography. Fundus and histology images of control mice (PBS) show severe inflammation characterized by blurred optic disc margins and enlarged juxtapapillary areas, papilledema, retinal vasculitis with moderate cuffing, vitreitis, retinal folds, substantial infiltration of inflammatory cells into the vitreous, choroiditis, and yellow-whitish retinal and choroidal infiltrates (Figures 2B,C). In contrast, images derived from fundus (Figure 2B) or histological (Figure 2C) analyses revealed mild EAU in eyes of mice treated with i35-Exosomes and clinical scores were significantly low compared to eyes of the untreated mice (Figure 2B; left panels). Optical coherence tomography (OCT) is a non-invasive procedure that allows visualization of internal microstructure of various eye structures in living animals and results of our OCT analysis revealed substantial accumulation of inflammatory cells in vitreous and optic nerve head of control untreated eyes compared to mice treated with i35-Exosomes (Figure 2D). Inflammation of the retina induces changes in the electroretinogram (ERG) indicative of alterations in visual function (18, 30) and it is assessed by recording changes in electrical potentials in response to light stimulation of the retina. ERG under light-adaptive stimuli reflects cone-driven signaling while the dark-adapted b-wave responses evaluate status of rod-driven signaling and lower a- and b-wave recordings are indicative of retinal pathology. EAU pathology is associated with defects in rod and cones attributed to attack of photoreceptor cells by inflammatory Th17 and/or Th1 cells (7, 31). We observed significant increase of a-wave and b-wave amplitudes in eyes with i35-Exosomes compared to the control eyes (Figure 2E), suggesting that defects in cone and rod signaling functions in normal mouse with EAU was rescued in part by i35-Exosome treatment. The observed defects in cone and rod signaling functions and higher EAU pathological score in untreated mice with EAU, suggest that i35-Exosomes contributed to mechanisms that prevented the decrement of visual impairment observed in mice during EAU.

### i35-Exosomes Suppress Th17 Responses During EAU by Inducing Expansion of Treg Cells

**Figure 3:**
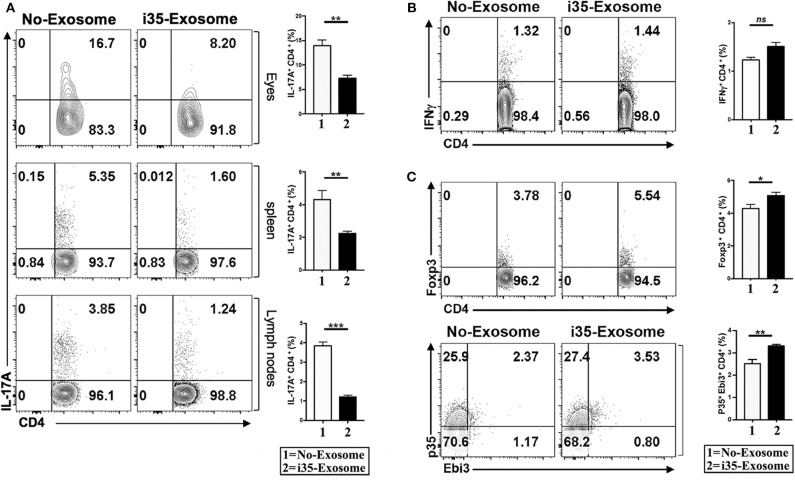
Amelioration of uveitis in i35-Exosome-treated mice correlates with suppression of Th17 responses and expansion regulatory T cells. **(A)** Analysis of CD4^+^ lymphocytes in the eye, spleen, or lymph nodes of mice treated with PBS or i35-Exosomes by the intracellular cytokine assay (*n* = *14*). **(B,C)** Analysis of CD4^+^ T cells in the eye of mice treated with PBS or i35-Exosomes by the intracellular cytokine assay (*n* = *14*). Data are presented as mean ± SEM of three replicates. Results represent three independent studies. *p < 0.05, **p < 0.01, ***p < 0.001.

EAU is a T cell mediated intraocular inflammatory disease and retinal pathology results in part from cytotoxic effects of proinflammatory cytokines secreted by inflammatory cells recruited into the retina during EAU. As Th1 and Th17 are implicated in the etiology of EAU (7, 31, 32), we investigated whether mechanistic basis for the suppression of EAU in mice treated with i35-Exosomes derived from antagonistic effects on proinflammatory responses. We induced EAU in C57BL/6J mice and fundoscopic examination of the eyes established that the development uveitis by day 15 post-immunization. The mice were then sacrificed on day 17 post-immunization and lymphocytes isolated from the retina, spleen, or lymph nodes were analyzed by the intracellular cytokine assay. Analysis of cells that infiltrate the eye during EAU revealed significant reduction of Th17 cells in eyes of mice treated with i35-Exosomes but not control mouse eyes (Figure 3A). Similarly, the levels of Th17 cells in the spleen or lymph nodes were markedly diminished providing suggestive evidence that i35-Exosomes antagonize Th17 responses during EAU (Figure 3A). Interestingly, analysis of the levels of Th1 cells in eyes of the EAU mice did not reveal significant difference between i35-Exosomes-treated and untreated mice (Figure 3B), suggesting that i35-Exosomes antagonized Th17 but not Th1 responses, consistent with previous reports showing that EAU pathology is mediated primarily by Th17 cells (33). As regulatory B cells (Bregs) and regulatory T cells (Tregs) have been shown to suppress pro-inflammatory responses that mediate uveitis (7, 9), we examined whether i35-Exosome-mediated attenuation of EAU derived in part from expansion of regulatory cells. We analyzed lymphocytes that infiltrate the eye during EAU and show here that Foxp3^+^ regulatory T cells and IL-35-producing regulatory T cells (iTR35) are significantly expanded (Figure 3C).

## Discussion

Uveitis is a diverse group of intraocular inflammatory diseases that includes birdshot retinochoroidopathy, Behcet's disease, ocular sarcoidosis and accounts for 10% of severe visual handicaps in the United States (21, 28). The disease can occur in the front of the eye (anterior uveitis), back of the eye (posterior uveitis) or all over the eye (pan uveitis) and may be of infectious or autoimmune etiology. Conventional treatment includes topical or systemic administration of corticosteroids. Although steroids are effective therapy for uveitis, serious adverse effects preclude their prolonged use. Biologics such as interferons, Tac antibody (Daclizumab), TNF-α blockers as well as slow-release ocular implants containing IL-10 provide viable alternatives to steroids in the treatment of recalcitrant, blinding ocular inflammatory diseases (34, 35). However, mechanisms underlying efficacy of these therapies have not been fully elucidated and considerable impetus is to develop alternative therapies such as biologics and cell-based therapies for uveitis.

Regulatory B-cells show substantial promise for cell therapy against autoimmune and neurodegenerative diseases. However, significant technically difficulties and labor intensive efforts required to manufacture sufficient quantities for therapeutic use, remain major obstacles to be overcome before they can be brought to the clinic. Moreover, Bregs suppress inflammation or autoimmune diseases in Ag-specific manner, restricting their suppressive effects to the specific autoantigen that elicits the disease. Nonetheless, i35-Breg-mediated suppression and amelioration of uveitis or encephalomyelitis in mouse models of human uveitis or multiple sclerosis is attributed to inhibitory effects of IL-35 secreted at inflammatory sites by i35-Bregs. In this study, we uncovered that Breg cells release exosomes that contain bioactive IL-35 (i35-Exosomes) and this may be an additional mechanism by which i35-Breg cells suppress inflammatory responses. However, we do not know whether release of i35-Exosomes is restricted to i35-Bregs or if all activated B cells can release i35-Exosomes.

EAU shares essential clinical features of human uveitis and provides a useful framework for evaluating therapies purported to suppress and/or ameliorate uveitis. EAU also shares essential immunopathogenic features with EAE, the animal model multiple sclerosis. Thus, we utilized the EAU model in this proof-of-principle study to demonstrate that i35-Bregs can be used to treat a CNS autoimmune disease. Indeed, i35-Exosomes suppressed EAU and conferred protection from ocular pathology by inhibiting the expansion and trafficking of pathogenic Th17 cells into the retina. ERG data showed that i35-Exosome rescued mice from decrement of retinal function associated with uveitis, underscoring the neuroprotective effect of i35-Exosome. Of clinical importance, i35-Exosome is non-toxic and mitigates uveitis without inducing systemic allogeneic immune responses, suggesting that i35-Exosome may complement anti-inflammatory agents currently used to treat uveitis. Although the focus of this study is on posterior uveitis, the most insidious form of uveitis, we envision that i35-Exosome may also be useful in suppressing anterior uveitis, cytokine-induced fibrosis during glaucoma surgery, as well as, limiting inflammation during ocular surgery or post-operatively after cataract surgery. Because exosomes readily cross the blood-retina-barrier, we are now developing topical preparations that can be administered as eye drops thereby obviating the need of intravenous administration. In our current study, treatment efficacy was achieved by administering i35-Exosome every day from day 9 to 14. For potential use of i35-Exosomes in human uveitis pharmacokinetic studies would be necessary to determine bioavailability of the i35-Exosome and minimal dose necessary to achieve therapeutic efficacy.

## Data Availability Statement

All datasets generated for this study are included in the article/supplementary material.

## Ethics Statement

The animal study was reviewed and approved by NIH Animal Care and Use Committee: approved animal Study Protocol # NEI-597.

## Author Contributions

MK performed experiments, prepared the figures and edited the manuscript. JC assisted with EAU induction, adoptive transfer studies, and analyzed FACS data. YJ assisted in EAU disease scoring and fundoscopy. CE conceived, designed, supervised project, and wrote manuscript.

## Conflict of Interest

The authors declare that the research was conducted in the absence of any commercial or financial relationships that could be construed as a potential conflict of interest.

## References

[1] Wraith DC Nicholson LB. The adaptive immune system in diseases of the central nervous system. J Clin Invest. (2012) 122:1172–9. 10.1172/JCI58648 22466659 PMC3314451

[2] Streilein JW. Ocular immune privilege: therapeutic opportunities from an experiment of nature. Nat Rev Immunol. (2003) 3:879–9. 10.1038/nri1224 14668804

[3] Wang X Wei Y Xiao H Liu X Zhang Y Han G. A novel IL-23p19/Ebi3 (IL-39) cytokine mediates inflammation in Lupus-like mice. Eur J Immunol. (2016) 46:1343–50. 10.1002/eji.201546095 27019190 PMC11334612

[4] Egwuagu CE Yu CR Sun L Wang R. Interleukin 35: critical regulator of immunity and lymphocyte-mediated diseases. Cytokine Growth Factor Rev. (2015) 26:587–93. 10.1016/j.cytogfr.2015.07.013 26279360 PMC4581966

[5] Sun L He C Nair L Yeung J Egwuagu CE. Interleukin 12 (IL-12) family cytokines: role in immune pathogenesis and treatment of CNS autoimmune disease. Cytokine. (2015) 75:249–55. 10.1016/j.cyto.2015.01.030 25796985 PMC4553122

[6] Vignali DA Kuchroo VK. IL-12 family cytokines: immunological playmakers. Nat Immunol. (2012) 13:722–8. 10.1038/ni.2366 22814351 PMC4158817

[7] Wang RX Yu CR Dambuza IM Mahdi RM Dolinska MB Sergeev YV. Interleukin-35 induces regulatory B cells that suppress autoimmune disease. Nat Med. (2014) 20:633–41. 10.1038/nm.3554 24743305 PMC4048323

[8] Choi JK Dambuza IM He C Yu CR Uche AN Mattapallil MJ. IL-12p35 inhibits neuroinflammation and ameliorates autoimmune encephalomyelitis. Front Immunol. (2017) 8:1258. 10.3389/fimmu.2017.01258 29051763 PMC5633738

[9] Dambuza IM He C Choi JK Yu CR Wang R Mattapallil MJ. IL-12p35 induces expansion of IL-10 and IL-35-expressing regulatory B cells and ameliorates autoimmune disease. Nat Commun. (2017) 8:719. 10.1038/s41467-017-00838-4 28959012 PMC5620058

[10] Tan L Wu H Liu Y Zhao M Li D Lu Q. Recent advances of exosomes in immune modulation and autoimmune diseases. Autoimmunity. (2016) 49:357–65. 10.1080/08916934.2016.1191477 27259064

[11] Saleh AF Lazaro-Ibanez E Forsgard MA Shatnyeva O Osteikoetxea X Karlsson F. Extracellular vesicles induce minimal hepatotoxicity and immunogenicity. Nanoscale. (2019) 11:6990–7001. 10.1039/C8NR08720B 30916672

[12] Okoye IS Coomes SM Pelly VS Czieso S Papayannopoulos V Tolmachova T. MicroRNA-containing T-regulatory-cell-derived exosomes suppress pathogenic T helper 1 cells. Immunity. (2014) 41:89–103. 10.1016/j.immuni.2014.05.019 25035954 PMC4104030

[13] Nazarenko I Rupp AK Altevogt P. Exosomes as a potential tool for a specific delivery of functional molecules. Methods Mol Biol. (2013) 1049:495–511. 10.1007/978-1-62703-547-7_37 23913240

[14] Batrakova EV Kim MS. Using exosomes, naturally-equipped nanocarriers, for drug delivery. J Control Release. (2015) 219:396–405. 10.1016/j.jconrel.2015.07.030 26241750 PMC4656109

[15] Li N Zhao L Wei Y Ea VL Nian H Wei R. Recent advances of exosomes in immune-mediated eye diseases. Stem Cell Res Ther. (2019) 10:278. 10.1186/s13287-019-1372-0 31470892 PMC6716826

[16] Shen P Roch T Lampropoulou V O'Connor RA Stervbo U Hilgenberg E. IL-35-producing B cells are critical regulators of immunity during autoimmune and infectious diseases. Nature. (2014) 507:366–70. 10.1038/nature12979 24572363 PMC4260166

[17] Mattapallil MJ Silver PB Cortes LM St. Leger AJ Jittayasothorn Y Kielczewski JL. Characterization of a new epitope of IRBP that induces moderate to severe uveoretinitis in mice with H-2b haplotype. Invest Ophthalmol Vis Sci. (2015) 56:5439–49. 10.1167/iovs.15-17280 26284549 PMC4544201

[18] He C Yu CR Sun L Mahdi RM Larkin J 3rd Egwuagu CE. Topical administration of a suppressor of cytokine signaling-1 (SOCS1) mimetic peptide inhibits ocular inflammation and mitigates ocular pathology during mouse uveitis. J Autoimmun. (2015) 62:31–8. 10.1016/j.jaut.2015.05.011 26094775 PMC4529792

[19] Oh HM Yu CR Lee Y Chan CC Maminishkis A Egwuagu CE. Autoreactive memory CD4+ T lymphocytes that mediate chronic uveitis reside in the bone marrow through STAT3-dependent mechanisms. J Immunol. (2011) 187:3338–46. 10.4049/jimmunol.1004019 21832158 PMC3304102

[20] Paques M Guyomard JL Simonutti M Roux MJ Picaud S Legargasson JF. Panretinal, high-resolution color photography of the mouse fundus. Invest Ophthalmol Vis Sci. (2007) 48:2769–74. 10.1167/iovs.06-1099 17525211

[21] Chan CC Caspi RR Ni M Leake WC Wiggert B Chader GJ. Pathology of experimental autoimmune uveoretinitis in mice. J Autoimmun. (1990) 3:247–55. 10.1016/0896-8411(90)90144-H 2397018

[22] Xu H Koch P Chen M Lau A Reid DM Forrester JV. A clinical grading system for retinal inflammation in the chronic model of experimental autoimmune uveoretinitis using digital fundus images. Exp Eye Res. (2008) 87:319–26. 10.1016/j.exer.2008.06.012 18634784

[23] Gabriele ML Ishikawa H Schuman JS Ling Y Bilonick RA Kim JS. Optic nerve crush mice followed longitudinally with spectral domain optical coherence tomography. Invest Ophthalmol Vis Sci. (2011) 52:2250–4. 10.1167/iovs.10-6311 21398282 PMC3080179

[24] Okoye IS Coomes SM Pelly VS Czieso S Papayannopoulos V Tolmachova T. MicroRNA-containing T-regulatory-cell-derived exosomes suppress pathogenic T helper 1 cells. Immunity. (2014) 41:503. 10.1016/j.immuni.2014.08.008 28903020 PMC5640441

[25] Wahlgren J Karlson Tde L Glader P Telemo E Valadi H. Activated human T cells secrete exosomes that participate in IL-2 mediated immune response signaling. PLoS One. (2012) 7:e49723. 10.1371/journal.pone.0049723 23166755 PMC3500321

[26] Wei X Zhang J Gu Q Huang M Zhang W Guo J. Reciprocal expression of IL-35 and IL-10 defines two distinct effector Treg subsets that are required for maintenance of immune tolerance. Cell Rep. (2017) 21:1853–69. 10.1016/j.celrep.2017.10.090 29141218

[27] Sawant DV Yano H Chikina M Zhang Q Liao M Liu C. Adaptive plasticity of IL-10(+) and IL-35(+) Treg cells cooperatively promotes tumor T cell exhaustion. Nat Immunol. (2019) 20:724–35. 10.1038/s41590-019-0346-9 30936494 PMC6531353

[28] Nussenblatt RB. Proctor Lecture. Experimental autoimmune uveitis: mechanisms of disease and clinical therapeutic indications. Invest Ophthalmol Vis Sci. (1991) 32:3131–41. 1748544

[29] Caspi RR Roberge FG Chan CC Wiggert B Chader GJ Rozenszajn LA. A new model of autoimmune disease. Experimental autoimmune uveoretinitis induced in mice with two different retinal antigens. J Immunol. (1988) 140:1490–5. 3346541

[30] Zimmermann N Mishra A King NE Fulkerson PC Doepker MP Nikolaidis NM. Transcript signatures in experimental asthma: identification of STAT6-dependent and -independent pathways. J Immunol. (2004) 172:1815–24. 10.4049/jimmunol.172.3.1815 14734765

[31] Amadi-Obi A Yu CR Liu X Mahdi RM Clarke GL Nussenblatt RB. T(H)17 cells contribute to uveitis and scleritis and are expanded by IL-2 and inhibited by IL-27/STAT1. Nat Med. (2007) 13:711–8. 10.1038/nm1585 17496900

[32] Luger D Silver PB Tang J Cua D Chen Z Iwakura Y. Either a Th17 or a Th1 effector response can drive autoimmunity: conditions of disease induction affect dominant effector category. J Exp Med. (2008) 205:799–810. 10.1084/jem.20071258 18391061 PMC2292220

[33] Liu X Lee YS Yu CR Egwuagu CE. Loss of STAT3 in CD4+ T cells prevents development of experimental autoimmune diseases. J Immunol. (2008) 180:6070–6. 10.4049/jimmunol.180.9.6070 18424728 PMC2435016

[34] Nussenblatt RB Fortin E Schiffman R Rizzo L Smith J Van Veldhuisen P. Treatment of noninfectious intermediate and posterior uveitis with the humanized anti-Tac mAb: a phase I/II clinical trial. Proc Natl Acad Sci U S A. (1999) 96:7462–6. 10.1073/pnas.96.13.7462 10377437 PMC22108

[35] Nussenblatt RB Thompson DJ Li Z Peterson JS Robinson RR Shames RS. Humanized anti-interleukin-2 (IL-2) receptor alpha therapy: long-term results in uveitis patients and preliminary safety and activity data for establishing parameters for subcutaneous administration. J Autoimmun. (2003) 21:283–93. 10.1016/j.jaut.2004.04.001 14599854

